# Robust Identification of Differential Gene Expression Patterns from Multiple Transcriptomics Datasets for Early Diagnosis, Prognosis, and Therapies for Breast Cancer

**DOI:** 10.3390/medicina59101705

**Published:** 2023-09-24

**Authors:** Khanis Farhana Tuly, Md. Bayazid Hossen, Md. Ariful Islam, Md. Kaderi Kibria, Md. Shahin Alam, Md. Harun-Or-Roshid, Anjuman Ara Begum, Sohel Hasan, Rashidul Alam Mahumud, Md. Nurul Haque Mollah

**Affiliations:** 1 Bioinformatics Lab, Department of Statistics, University of Rajshahi, Rajshahi 6205, Bangladesh; farhanatuly119@gmail.com (K.F.T.); bayazid.stat@gmail.com (M.B.H.); ariful.stat.bio@gmail.com (M.A.I.); kibriastat15@gmail.com (M.K.K.); shahin4824@gmail.com (M.S.A.); harun.stat.ru@gmail.com (M.H.-O.-R.); aab_stat@yahoo.com (A.A.B.); 2 Department of Statistics, Hajee Mohammad Danesh Science & Technology University, Dinajpur 5200, Bangladesh; 3 Molecular and Biomedical Health Science Lab, Department of Biochemistry and Molecular Biology, University of Rajshahi, Rajshahi 6205, Bangladesh; sohel_bio@yahoo.com; 4 NHMRC Clinical Trials Centre, Faculty of Medicine and Health, The University of Sydney, Camperdown, NSW 2006, Australia; rashed.mahumud@sydney.edu.au

**Keywords:** breast cancer, transcriptomics profiles, hub-genes, early diagnosis, prognosis and therapies, integrated robust statistics and bioinformatics approaches

## Abstract

*Background and Objectives:* Breast cancer (BC) is one of the major causes of cancer-related death in women globally. Proper identification of BC-causing hub genes (HubGs) for prognosis, diagnosis, and therapies at an earlier stage may reduce such death rates. However, most of the previous studies detected HubGs through non-robust statistical approaches that are sensitive to outlying observations. Therefore, the main objectives of this study were to explore BC-causing potential HubGs from robustness viewpoints, highlighting their early prognostic, diagnostic, and therapeutic performance. *Materials and Methods:* Integrated robust statistics and bioinformatics methods and databases were used to obtain the required results. *Results:* We robustly identified 46 common differentially expressed genes (cDEGs) between BC and control samples from three microarrays (GSE26910, GSE42568, and GSE65194) and one scRNA-seq (GSE235168) dataset. Then, we identified eight cDEGs (*COL11A1*, *COL10A1*, *CD36*, *ACACB*, *CD24*, *PLK1*, *UBE2C*, and *PDK4*) as the BC-causing HubGs by the protein-protein interaction (PPI) network analysis of cDEGs. The performance of BC and survival probability prediction models with the expressions of HubGs from two independent datasets (GSE45827 and GSE54002) and the TCGA (The Cancer Genome Atlas) database showed that our proposed HubGs might be considered as diagnostic and prognostic biomarkers, where two genes, *COL11A1* and *CD24*, exhibit better performance. The expression analysis of HubGs by Box plots with the TCGA database in different stages of BC progression indicated their early diagnosis and prognosis ability. The HubGs set enrichment analysis with GO (Gene ontology) terms and KEGG (Kyoto Encyclopedia of Genes and Genomes) pathways disclosed some BC-causing biological processes, molecular functions, and pathways. Finally, we suggested the top-ranked six drug molecules (Suramin, Rifaximin, Telmisartan, Tukysa Tucatinib, Lynparza Olaparib, and TG.02) for the treatment of BC by molecular docking analysis with the proposed HubGs-mediated receptors. Molecular docking analysis results also showed that these drug molecules may inhibit cancer-related post-translational modification (PTM) sites (Succinylation, phosphorylation, and ubiquitination) of hub proteins. *Conclusions*: This study’s findings might be valuable resources for diagnosis, prognosis, and therapies at an earlier stage of BC.

## 1. Introduction

Breast cancer (BC) is one of the most deadly cancers and malignant tumors for women worldwide [1]. It happens when the breast cells grow and divide in an uncontrolled way and create a mass of tissue called a tumor. The symptoms of BC tumors may include feeling a lump in the breast and a change in breast size and skin. In 2020, there were more than 2.3 million new cases and 685,000 BC mortalities. Thus, it has evolved into a major public health issue across the world that has created serious financial stress on patients and families. Despite the fact that the overall survival ratio of BC patients is quite poor, there is a strong chance of their recovery if it is diagnosed and treated properly in the early stages [2,3,4]. In spite of high incidence rates, the 5-year relative survival rate of diagnosed BC patients is about 90% in Western countries as well as in developed Asian countries [5]. However, the molecular mechanisms of BC initiation and progression are still unclear to the researchers in some cases. There are several non-causal risk factors (drinking alcohol, obesity, diabetes, lack of physical activity, early age at first menstruation, hormone replacement therapy after menopause, elderly age, late pregnancy, and family background) that do not directly cause BC but may increase a person’s risk of developing it indirectly or in association with other factors [6,7]. Generally, dysregulated hub genes (HubGs) are considered the causal risk factors for cancer. These genes are known as the differentially expressed genes (DEGs) between cancer and normal (control) samples. Genes may be dysregulated because of their DNA mutation, methylation, or other epigenetic factors. The dysregulated genes that are associated with cancer incidence are also known as oncogenes (upregulated DEGs) and tumor suppressor genes (downregulated DEGs) [8,9,10]. New drug discovery is a challenging and difficult task that is expensive, laborious, and time-consuming. Repurposing existing drugs reduces time and cost compared to de-novo drug discovery. However, in both cases, disease-causing HubGs-mediated receptor proteins play a vital role in exploring candidate drug molecules [11,12,13,14,15]. Therefore, the detection of BC-causing potential HubGs is essential for the diagnosis, prognosis, and therapies of BC at the earlier stages.

**Figure 1:**
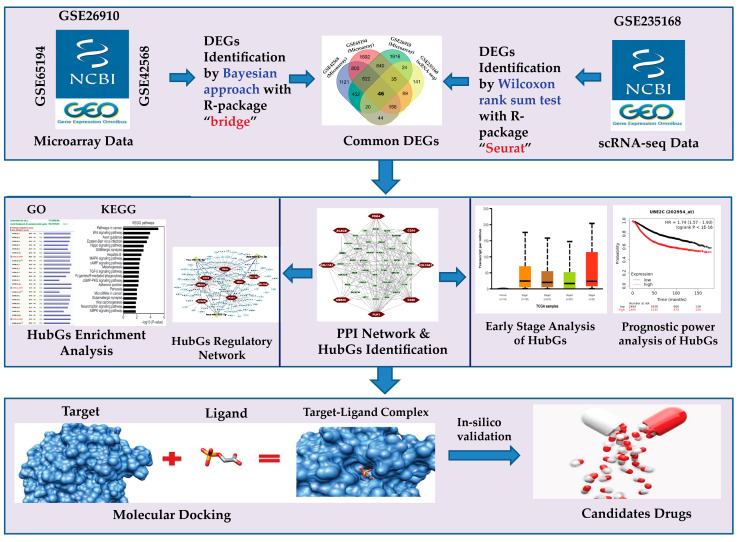
Pipeline of this study.

There are several individual studies in the literature that suggest BC-causing HubGs from DEGs. We reviewed 59 individual articles that recommend BC-causing HubGs sets. We observed that those HubGs sets are not so consistent (see Table S1 in the Supplementary File). We found a total of 286 different HubGs in those articles and observed that there were no common HubGs in those articles. It may be occurred because of regional/environmental variations with the sample units or the application of non-robust statistical methods for the identification of DEGs, since transcriptomics datasets (microarrays, RNA-seq counts) often suffer from few outlying observations due to the different steps required for data generation [16,17,18,19,20,21,22]. To explore DEGs, most of the studies considered the LIMMA approach [1,14,23,24,25,26,27,28,29,30,31,32,33,34,35,36,37,38,39,40,41,42,43,44,45,46,47,48,49,50,51,52,53,54,55,56,57,58,59,60,61,62,63,64,65,66,67,68]. Some studies considered SAM [68,69], *t*-test [70,71], WGCNA [72,73], and some other tools [66,67,74,75,76,77,78,79] for detecting DEGs between BC and control samples. However, most of these techniques, including LIMMA, SAM, t, and WGCNA, are sensitive to outlying observations, for which sometimes they produce misleading results [16,17,18,19,20,21,22]. On the other hand, most of them did not investigate the diagnosis and prognosis performance of their HubGs at the earlier stages, computationally or experimentally. Therefore, this study aimed to explore BC-causing potential HubGs from robustness viewpoints, highlighting their early prognostics, diagnostics, and therapeutic performance by using integrated statistics and bioinformatics approaches. The overview of this study is given in Figure 1.

## 2. Materials and Methods

### 2.1. Data Collection

In this study, we collected two types of transcriptomics data (microarray and single-cell RNA sequence (scRNA-seq) count data) to explore BC-causing HubGs. Then, we collected meta-drug agents from online sources to explore candidate drug agents as inhibitors of HubGs. The detailed descriptions of these datasets are given below.

#### 2.1.1. Microarray Data Collection

Three microarray transcriptomics datasets of breast cancer were collected from the NCBI-GEO database with accession numbers GSE26910, GSE42568, and GSE65194 under the platform GPL570 Affymetrix Human Genome U133 Plus 2.0 Array [80]. The GSE26910 dataset contained 6 samples of invasive breast and 6 normal samples; GSE42568 contained 104 BC samples and 17 normal breast samples; and GSE65194 contained 130 BC samples and 11 normal breast samples.

#### 2.1.2. The scRNA-seq Count Data Collection and Pre-Processing

The scRNA-seq dataset (GSE235168 [81]) was obtained from the publicly available NCBI GEO database [80]. The data were sequenced on Illumina HiSeq 4000 (human) and NextSeq 2000 (human). The dataset was pre-processed using the Seurat R package [82].

#### 2.1.3. Collection of Meta-Drug Agents

To select the potential drug agents, a total of 142 BC-associated meta-drug agents were collected from published articles and the online database GSCALite [83] (see Table S2).

### 2.2. Identification of DEGs from Microarray Data

The Bayesian robust inference for differential gene expression (BRIDGE) [20] approach with R-package ‘bridge’ was used to robustly identify DEGs between BC and control samples from three microarray datasets (GSE26910, GSE42568, and GSE65194), separately. We selected the DEGs by applying the posterior probability (PostP) and median fold change (mFC) based criterion with the cutoff at PostP ≥ 0.50 and |Log_2_(mFC)| ≥ 0.5, where mFC represents the median of FC values [21]. We separated the up- and down-regulated DEGs by applying the following criterion.

$${DEG}_{g}=\left\{\begin{array}{c} Upregulated,\;\mathrm{if}\;{PostP}_{g}\geq 0.50\;\mathrm{and}\;{{Log}_{2}(mFC}_{g})\geq 0.5\;\\ Downregulated,\;if\;{PostP}_{g}\geq 0.50\;\mathrm{and}\;{{Log}_{2}(mFC}_{g})\leq -0.5 \end{array}\right.$$

for *g*th gene (*g* = 1,2,…*G*), where *G* is the total number of genes.

### 2.3. Identification of DEGs from scRNA-seq Count Data

To identify DEGs from scRNA-seq count data (GSE235168), an R package, Seurat [82] was used with the cutoff at adj. *p-*value < 0.05 and |logFC| > 0.5. The Seurat [82] R-package utilized the non-parametric Wilcoxon rank sum test [84] to calculate the *p*-values from the robustness viewpoints.

### 2.4. Identification of Common DEGs (cDEGs)

We identified cDEGs as the BC-causing genes by taking common among the four DEGs sets, where three DEGs sets were detected from three microarray datasets and the rest, one DEGs set, was identified from the scRNA-seq count dataset, and visualized them by the Venn diagram.

### 2.5. Protein-Protein Interaction (PPI) of cDEGs for Identification of Hub Gene (HubGs)

Protein-protein interaction (PPI) occurs within the cell to perform its essential functions. The information produced by PPI networks helps us detect key genes [85]. Using the STRING (Search Tool for the Retrieval of Interacting Genes) database, a PPI-network of cDEGs was created for identifying HubGs [86]. To create the PPI network, the distance “*D*” between any two proteins, denoted as *A* and *B*, is computed as follows:

$$D\left(A,B\right)=\frac{2\;|N_{A}\cap N_{B}|}{\left|N_{A}\right|+|N_{B}|}$$

where $N_{A}$ and $N_{B}$ are the neighbor sets of u and v, respectively.

We used the Cytoscape software (Version 3.8.1) to increase the quality of the PPI network [87]. The cytoHubba package plugin in Cytoscape was used to select HubGs. Three topological measures, including degree, closeness, and betweenness, were used to select the HubGs.

### 2.6. HubGs-Set Enrichment Analysis with GO-Terms and KEGG Pathways

The enrichment analysis with GO (gene ontology) terms is performed in three categories: MFs (molecular functions), BPs (biological processes), and CCs (cellular [88] components). The GO terms are investigated for a better understanding of molecular mechanisms, cellular function, and sub-cellular locations where genes perform their functions. The KEGG (Kyoto Encyclopedia of Genes and Genomes) pathways are investigated to understand metabolic pathways and make extensive gene annotations [89]. The Enrichr [90] web tool was applied to select significantly enriched GO-terms and KEGG pathways by using the Fisher exact test procedure based on hypergeometric distribution [21]. The HubGs-set was significantly enriched with a GO-term/KEGG-pathway at the 5% level of significance if it’s adjusted *p*-value (*p_i_*) < 0.05. We adjusted the *p*-values by using the Benjamini and Hochberg procedures.

### 2.7. HubGs Regulatory Network Analysis

To identify the key transcription factors (TFs) and microRNAs (miRNAs) that regulate HubGs, we analyzed their interactions with HubGs based on the JASPAR [91] and TarBase v8.0 0 [92] databases through the NetworkAnalyst’s [93] web tool. The Cytoscape software [87] was considered to be a better representation of the networks.

### 2.8. Prognostic Performance of HubGs with the Independent Datasets

To assess the prognostic performance of HubGs, we investigated their survival and prediction performances with independent datasets. We performed disease-free survival (DFS) and progression-free survival (PFS) analysis on BC patients with the expressions of 8 HubGs by using the TCGA data-based “Kaplan-Meier plotter” (https://kmplot.com/) [94] and the “OSbrca” (https://bioinfo.henu.edu.cn/BRCA/BRCAList.jsp) [95] web-tools (accessed on 23 August 2023), respectively. The significant difference in survival probability curves for the high and low expression groups was investigated by hazard ratio (HR) and log-rank tests. The cut-off *p-*value < 0.05 was used in this study for statistical significance. We also investigated the expression levels of HubGs with an independent TCGA [96] and Genotype-Tissue Expression (GTEx) Portal [97] database using the GEPIA2 [98] web tool and constructed box plots for the expression levels of HubGs in BC tissues and compared them to those in normal tissues. Then, we investigated their prediction performance with a Random Forest (RF)-based prediction model. To implement the prediction, we transformed microarray datasets using the following robust formula:

$$X_{ij}^{*}=\frac{X_{ij}-med(X_{i})}{{IQR}_{i}}$$

where, $X_{ij}$ stands for the expression value of *i*th HubGs in the *j*th sample; $med(X_{i})$ and ${IQR}_{i}$ stand for the median and interquartile range of $i^{th}$ HubGs, respectively. We computed the prediction performance scores with the two independent test datasets with NCBI accession numbers, GSE45827 and GSE54002, for BC and control samples. We visualized the prediction performance by constructing ROC curves using the R package “ROCR” [99].

### 2.9. Expression Analysis of HubGs in Different BC Development Stages

To investigate the performance of HubGs as diagnostic and prognostic biomarkers at the earlier stages, we produced Box plots based on the expression levels of HubGs in different BC development stages (control group, stages-1, stage-2, stage-3, and stage-4) by using the UALCAN web-tool with the TCGA database [100,101].

### 2.10. HubGs-Guided Drug Repurposing

To repurpose effective drug molecules for the treatment of BC, molecular docking analysis of HubGs-mediated receptor proteins with different drug agents was performed. For docking analysis, different drug molecules (ligands/agents) were collected from different sources, as mentioned in Section 2.1.3. The molecular docking interaction requires 3D (3-Dimensional) structures of both target proteins and drug agents. The 3D structure of all target protein receptors was downloaded from the Protein Data Bank (PDB) [102], AlphaFold Protein Structure Database [103] and SWISS-MODEL [104]. The drug agent’s 3D structure was collected from the PubChem [105] database. The protein was preprocessed by removing the water molecules, and ligand heteroatoms and polar hydrogens were added using AutoDock Tools 1.5.7 [106]. Further, the drug ligands were prepared for docking simulation by setting the torsion tree through AutoDock Tools 1.5.7. AutoDock Vina [107] was used for calculating the binding affinities between target proteins and drug ligands. The criterion for exhaustiveness was set to 8. Discovery Studio Visualizer 2020 [108] was used to explore the distances and types of non-covalent bonds, docked complexes, and surface complexes. Let the binding score between the *i*th receptor and the *j*th ligand be S*_ij_* (*i* = 1, 2, … *m*, *j* = 1, 2, … *n*). Then, both the receptors and ligands are arranged in descending order of row and column means of the score matrix $S=\left[S_{ij}\right]$ to select the top-ranking few drugs as the candidate drug molecules. We verified the inhibiting power of our suggested drug molecules against the top-ranked BC-causing HubGs suggested by other individual studies. We also verified the binding performance of the selected drug molecules against the cancer-related PTM (post-translational modification) sites of hub proteins through the docking analysis [109,110,111,112,113].

## 3. Results

### 3.1. Identification of DEGs from Microarray Data

We identified three DEG sets from three microarray transcriptomics datasets with NCBI-GEO accession IDs GSE26910, GSE42568, and GSE65194, respectively, by using the statistical BRIDGE and median fold change (mFC) approaches from the robustness viewpoints as described previously. From the GSE26910 dataset, we identified 555 DEGs with 367 down-regulated and 188 up-regulated genes. In GSE42568, there were 3164 DEGs, of which 1654 were down-regulated and 1510 were up-regulated. In GSE65194, we found 5256 DEGs, of which 1715 were down-regulated and 3541 were up-regulated.

### 3.2. Identification of DEGs from scRNA-seq Count Data

To identify DEGs from the scRNA-seq count dataset, we analyzed the dataset with NCBI accession ID GSE235168 and found a total of 3923 DEGs by applying the Seurat R package, as described previously.

### 3.3. Identification of Common DEGs (cDEGs)

**Figure 2:**
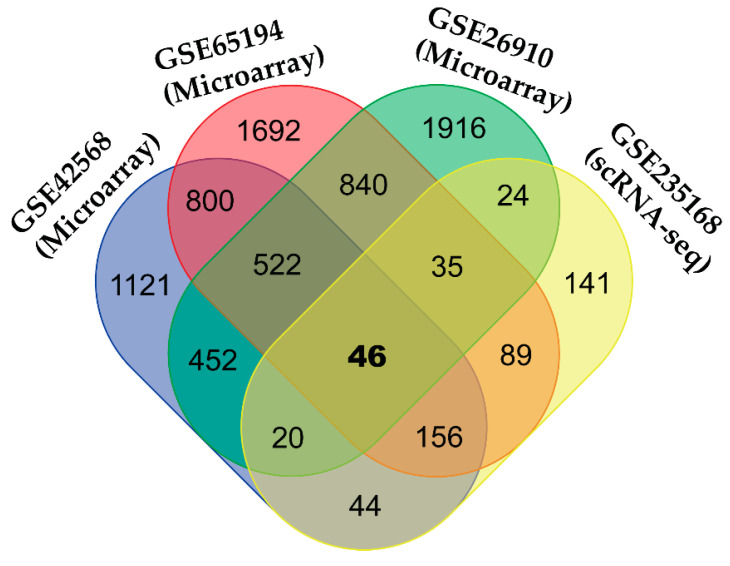
Venn diagrams for common DEGs among the four DEGs sets. Different colors indicate different numbers of DEGs for different combinations of four DEGs sets.

We identified 46 cDEGs, including 18 up-regulated (CD24, BGN, GALNT10, CXCL10, NREP, COL11A1, CTTN, JPT1, IDH2, EPPK1, FN1, INHBA, SDC1, FAM83D, COL10A1, TRIM59, PLK1, UBE2C) and 28 down-regulated (NFIA, EIF1, SPTBN1, RBP7, PDK4, TACC1, CXCL2, PALMD, ADIRF, TFPI, AKR1C1, ACACB, NOVA1, ABCA5, PCOLCE2, SYNM, CD36, PLAGL1, NR3C1, SLIT3, ARRB1, PTX3, MGLL, ADAMTS1, LIFR, RASD1, SOCS2, FAM107A) as the BC-causing genes from three microarrays and one scRNA-seq count data and visualized them by the Venn diagram (see Figure 2).

### 3.4. PPI Network of cDEGs for Identification of HubGs

**Figure 3:**
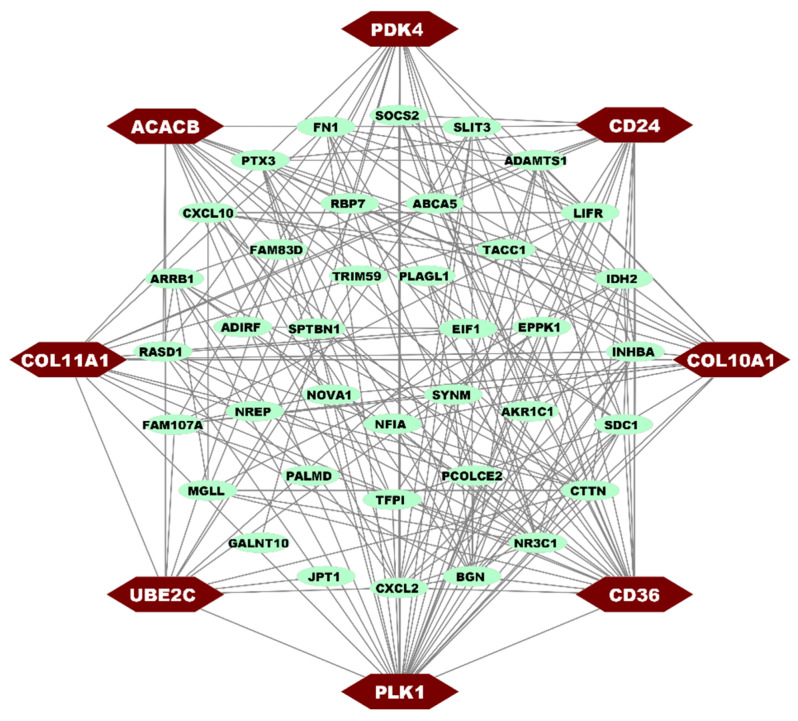
Protein-Protein interaction of cDEGs A hexagon with a red color represents the 8 HubGs, and a green color represents other DEGs.

A protein-protein interaction (PPI) network of DEGs was constructed using the STRING database. It was constructed with 46 nodes and 196 edges. We used three topological measures (degree, closeness, and betweenness) in cytoHubba to select HubGs, as displayed in Table 1. Taking the union of the top-ranked 5 genes with each measure, we obtained 8 cDEGs, including 5 upregulated (*COL11A1*, *COL10A1*, *CD24*, *PLK1*, *UBE2C*) and 3 downregulated (*PDK4*, *CD36*, *ACACB*) as the BC-causing hub genes (HubGs) that are displayed in the PPI network (see Figure 3).

### 3.5. HubGs-Set Enrichment Analysis with GO-Terms and KEGG Pathway

HubGs-set enrichment analysis with GO-terms (BPs, MFs, CCs) revealed that HubGs-set is significantly enriched in Regulation of Ubiquitin Protein Ligase Activity with HubGs (UBE2C and PLK1), Regulation of Mitotic Cell Cycle Phase Transition with HubGs (UBE2C and PLK1), Extracellular Structure Organization with HubGs (COL11A1 and COL10A1), and so on in other BPs. In CCs, Membrane Raft with HubGs (CD36 and CD24), Endoplasmic Reticulum Lumen with HubGs (COL11A1 and COL10A1), Platelet Alpha Granule Membrane with HubGs (CD36), and so on For MFs, Protein Tyrosine Kinase Activator Activity with HubGs (CD36 and CD24), ATP Binding with HubGs (PLK1 and PDK4), Ubiquitin Conjugating Enzyme Activity with HubGs (UBE2C), and so on (Table 2). In the case of KEGG pathways, Adipocytokine signaling pathway with HubGs (CD36 and ACACB), Protein digestion and absorption with HubGs (COL11A1 and COL10A1), Insulin resistance with HubGs (CD36 and ACACB), AMPK signaling pathway with HubGs (CD36 and ACACB), and Fatty acid biosynthesis with HubGs (ACACB), as displayed in Table 2.

### 3.6. HubGs Regulatory Network Analysis

**Figure 4:**
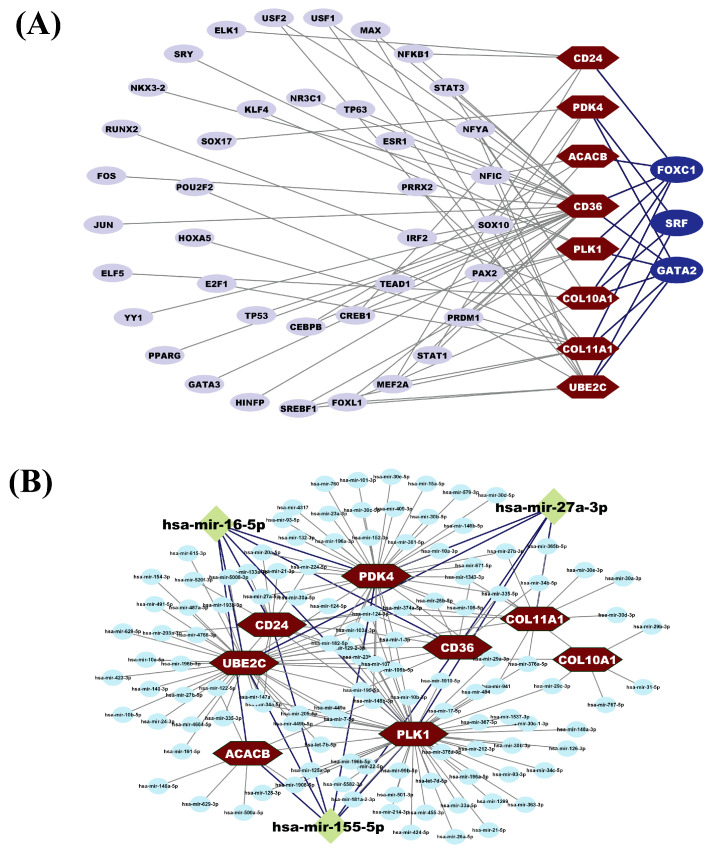
HubGs regulatory networks (**A**) TFs-HubGs interaction network. A hexagon with dark red indicates HubGs, an ellipse with blue indicates key TF proteins, and a light blue indicates other TF proteins. (**B**) miRNA-HubGs interaction network. A diamond with a green color indicates key miRNAs, and a circle with a sky blue color indicates other miRNAs.

The HubGs-TFs interaction network was constructed by using NetworkAnalyst in the JASPAR database to detect the transcriptional regulators of HubGs. Based on the higher degree of connectivity with HubGs, we selected three TF proteins (FOXC1, GATA2, and SRF) as the crucial transcriptional regulators of HubGs (Figure 4A). We observed that FOXC1 is the transcriptional regulator of 6 HubGs (*COL11A1*, *COL10A1*, *CD36*, *ACACB*, *CD24*, and *PLK1*), GATA2 is the transcriptional regulator of 6 HubGs (*COL11A1*, *COL10A1*, *CD36*, *UBE2C*, *PLK1*, and *PDK4*), and SRF is the transcriptional regulator of 3 HubGs (*COL11A1*, *COL10A1*, and *PDK4*). Moreover, the HubGs-miRNA interaction network was constructed by using NetworkAnalyst in the TarBase database to detect the post-transcriptional regulators of HubGs. We selected three miRNAs (hsa-mir-16-5p, hsa-mir-155-5p, and hsa-mir-27a-3p) as the key post-transcriptional regulators of HubGs (Figure 4B). The hsa-mir-16-5p, which regulates *ACACB*, *CD24*, *PLK1*, *UBE2C*, *CD36*, and *PDK4*, was selected based on its betweenness score of 436.23. Similarly, hsa-mir-155-5p, regulating *ACACB*, *CD24*, *PLK1*, *UBE2C*, *CD36*, and *PDK4*, also had a betweenness score of 436.23. Additionally, hsa-mir-27a-3p, a regulator of *PLK1*, *UBE2C*, *COL11A1*, *CD36*, and *PDK4*, was selected with a betweenness score of 366.63.

### 3.7. Prognostic Performance of HubGs with the Independent Expression Datasets

To assess the prognostic performance of HubGs with the independent datasets, we performed disease-free survival (DFS) and progression-free survival (PFS) analyses, boxplot analyses, and an RF-based prediction model.

**Figure 5:**
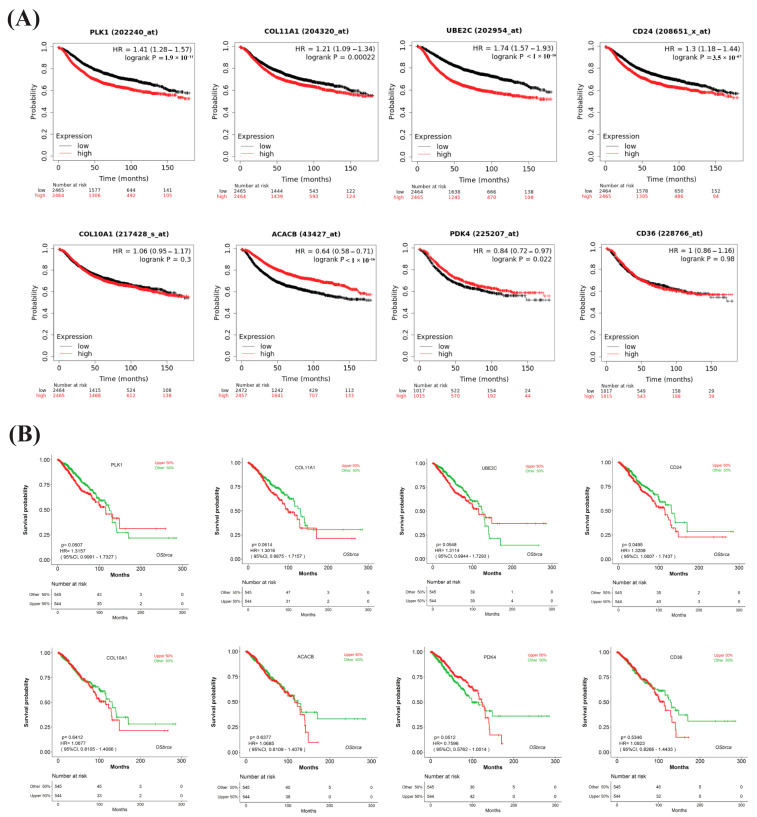
Prognostic performance of HubGs (**A**) Disease-free survival (DFS) probability curves for the low (black) and high (red) expression groups of BC patients based on the expressions of HubGs. (**B**) Progression-free survival (PFS) probability curves for the low (green) and high (red) expression groups of BC patients based on the expressions of HubGs.

From the survival probability curves, we observed that higher expressions of two upregulated HubGs (*COL11A1* and *CD24*) exhibit lower DFS and PFS probabilities compared to their lower expressions, which satisfy the expected impact on survival. Another three upregulated HubGs (*PLK1*, *UBE2C*, and *COL10A1*) and two downregulated HubGs (*PDK4* and *ACACB*) partially supported the expected impact on survival. The remaining downregulated gene, *CD36*, did not significantly satisfy the expected outcome (see Figure 5A,B). We also verified the expression patterns of HubGs by the independent TCGA and GTEx databases and found that three HubGs (*PDK4*, *ACACB*, and *CD36*) were downregulated and the rest five HubGs (*COL11A1*, *COL10A1*, *CD24*, *PLK1*, and *UBE2C*) were up-regulated, which supports our previous results (see Figure S1). To assess prediction performance by HubGs, we developed a random forest (RF)-based prediction model with the expressions of HubGs by using 60% of BC and control samples. Then, we calculated the prediction performance scores for the test dataset, which was created using the remaining 40% of the samples. We also computed the prediction performance with two independent test datasets (GSE45827 and GSE54002). We observed that the prediction model exhibited good performance for both the test and independent test datasets, with an AUC (Area Under the ROC Curve) exceeding 0.96 and an ACC (Accuracy) greater than 0.88 (Figure S2 and Table S3).

### 3.8. Expression Analysis of HubGs in Different BC Development Stages

The box-plot analysis based on the expressions of HubGs in different BC development stages (control group, stages-1, stage-2, stage-3, and stage-4) was performed to investigate the performance of HubGs as early diagnostic and prognostic biomarkers.

**Figure 6:**
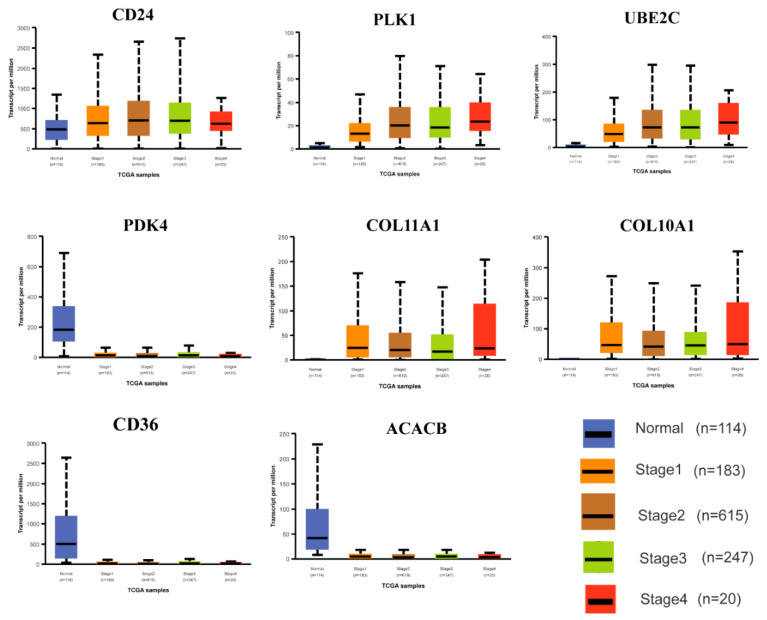
Assessment of HubGs by Box plots in different stages of BC.

Among the eight HubGs, three (*CD36*, *ACACB*, and *PDK4*) were found to be down-regulated genes in different stages of BC progression, and the rest five HubGs (*COL11A1*, *COL10A1*, *PLK1*, *UBE2C*, and *CD24*) exhibited upregulation in different stages of BC progression compared to the control group. However, in the case of the *CD24* gene, it showed an upregulation trend up to stage 3, slightly decreased in stage 4, but still remained upregulated compared to the control group (Figure 6). So, our proposed HubGs may be utilized as strong diagnostic and prognostic biomarkers at an earlier stage of BC.

### 3.9. HubGs-Guided Drug Screening by Molecular Docking

**Figure 7:**
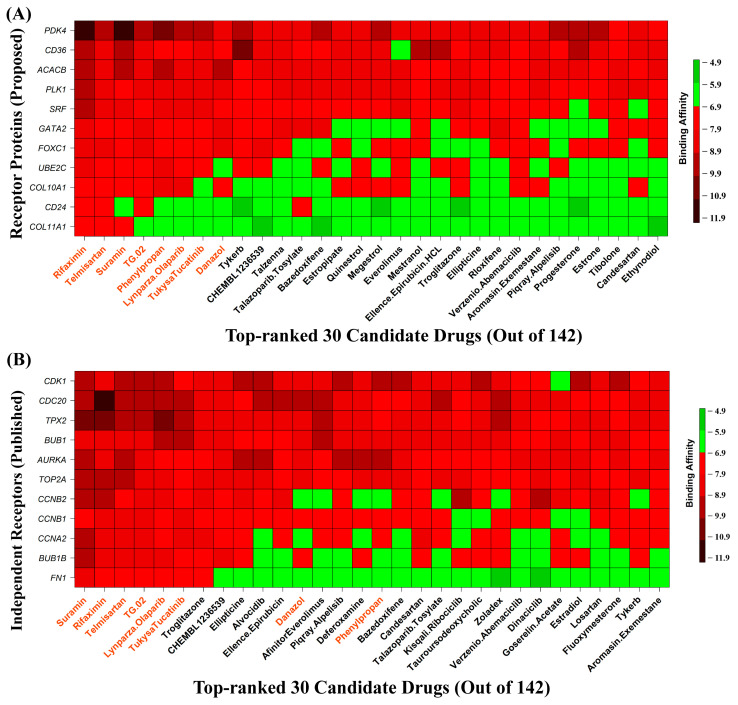
In the results of the molecular docking simulation, a red color indicates strong bindings, and a green color indicates weak bindings. (**A**) The image of binding affinity scores of 11 proposed proteins with the top-ordered 30 drug agents out of 142. (**B**) The image of binding affinity scores of 11 published proteins with the top-ordered 30 drug agents out of 142.

To explore candidate drug molecules for BC, we considered 8 HubGs-mediated proteins (COL11A1, COL10A1, CD36, ACACB, CD24, PLK1, UBE2C, and PDK4) and 3 regulatory TF proteins (GATA2, SRF, and FOXC1) as the receptors for docking analysis with 142 candidate drug molecules (ligands) that were collected from different sources (see Table S2). The 3D structures of COL11A1, COL10A1, CD36, ACACB, PDK4, CD24, PLK1, UBE2C, FOXC1, GATA2, and SRF were downloaded from PDB, SWISS-MODEL, and AlphaFold Protein Structure Database with codes P12107, 1GR3, 5LGD, 2DN8, 2ZDX, A0A151MJG4, 1Q4K, O00762, Q12948, 6ZFV, and P11831, respectively. The PubChem database was used for downloading the 3D structures of ligands. Then, we applied molecular docking simulations between our proposed HubGs-mediated receptors and meta-ligands. We construct a matrix based on the binding affinity scores. HubGs/receptors were ordered in rows of the matrix based on the average binding affinity scores across the candidate ligands. Similarly, ligands were arranged in the columns of the matrix based on the average binding affinity scores across the HubGs (Figure 7A). We observed that the top-order eight ligands are Suramin, Rifaximin, Telmisartan Phenylpropan, Tukysa Tucatinib, Lynparza Olaparib, TG.02, and Danazol, with average binding affinity scores across the receptors of less than −7.7 (kcal/mol). To verify their binding performance compared to the rest of the 142 ligands suggested by others, we also performed molecular docking analysis of the top-ranked 11 independent receptors (AURKA, BUB1, FN1, TPX2, CDC20, CCNA2, CCNB2, CCNB1, BUB1B, CDK1, and TOP2A) suggested by others with the aforementioned 142 ligands. The 3D structure of 10 independent receptors, AURKA, BUB1, FN1, TPX2, CDC20, CCNA2, CCNB1, BUB1B, CDK1, and TOP2A, was downloaded from the PDB database with the codes 5G1X, 6F7B, 1FNA, 6VPM, 4GGC, 6ATH, 5LQF, 2WVI, 6GU2, and 5NNE, respectively, and CCNB2 from SWISS-MODEL with UniProt ID O95067. Figure 7B displays the ordered independent receptors in rows and the ordered ligands in columns based on their total binding affinities. In both Figure 7A,B, we observed that six ligands (Suramin, Rifaximin, Telmisartan, Tukysa Tucatinib, Lynparza Olaparib, and TG.02) are common in the top-ranked eight ligands. Therefore, in this study, we suggested these six ligands for the treatment against BC, in which three ligands (Suramin, Rifaximin, and Telmisartan) stay in the top-order three positions in both cases, and they bind all receptors strongly and significantly, with average binding affinity scores across all the receptors less than −9.5 (kcal/mol).

Different types of post-translational modifications (PTMs) of proteins are associated with different types of cancer. Some studies reported that the phosphorylation, succinylation, and ubiquitination sites of proteins are the BC-causing PTM sites [110,112,113,114]. Therefore, we verified the resistance power of the top-ranked three ligands (Suramin, Rifaximin, and Telmisartan) against the phosphorylation, succinylation, and ubiquitination sites of BC-causing HubGs-mediated receptor proteins by molecular docking analysis. We predicted the succinylated sites of our proposed receptor proteins by using the web-based prediction model SuccinSite [109] and the phosphorylated and ubiquitinated sites by MusiteDeep [111]. Table 3 and Supplementary Tables S4–S6 showed the binding affinity scores of our proposed ligands with different PTM sites (phosphorylation, succinylation, and ubiquitination). We observed that most of the binding affinity scores are significantly higher, which indicates our proposed ligands seem to be effective against various post-translational modification (PTM) sites of BC-causing hub proteins.

Some basic information regarding the complexes of top-ordered three ligands (Suramin, Rifaximin, and Telmisartan) and top-ranked three receptor proteins (ACACB, PDK4, and CD36) interactions, including 3D view, protein and ligand interaction, interacting amino acid residues, and bond type, is displayed in Table 4.

## 4. Discussion

In this study, we robustly identified 46 common DEGs (cDEGs) between Breast Cancer (BC) and control samples from three microarray gene-expression datasets (GSE26910, GSE42568, and GSE65194) and one scRNA-seq count dataset (GSE235168). Out of 46 cDEGs, we identified eight top-ranked hub genes (HubGs), which consisted of three downregulated DEGs (*CD36*, *ACACB*, and *PDK4*) and five upregulated DEGs (*COL11A1*, *COL10A1*, *CD24*, *PLK1*, and *UBE2C*). We also verified these HubGs as the BC-causing genes through the literature review. Among the identified HubGs, *COL11A1* (Collagen Type XI Alpha 1) and *COL10A1* (Collagen Type X Alpha 1 Chain) belong to the collagen family of proteins that is responsible for constituting essential structural components of the extracellular matrix (ECM), featuring domains with a distinctive triple-helical conformation [115]. Some previous studies found that *COL11A1* and *COL10A1* were upregulated in BC [116,117,118,119] and could serve as prospective biomarkers and/or novel drug targets for BC [120]. The gene *CD36* (cluster of differentiation 36) is a pivotal cell surface scavenger receptor in various cancers, including breast, brain, and ovarian, engaging in multifaceted functions such as fatty acid uptake, cellular adhesion, immune response, and apoptosis regulation within diverse cellular and environmental contexts. Due to its varied function in tumor biology, *CD36* has rapidly become an appealing therapeutic target in cancer [121]. In BC patients, *CD36* expression increases following anti-HER2 therapy, which relates to a low prognosis [122]. In addition, *CD24* (cluster of differentiation 24) is a glycosylated mucin-like antigen, found on cell surfaces. It tends to be more elevated in breast cancer compared to normal breast tissue and is linked to a poorer prognosis [123]. Previous analysis demonstrated that *ACACB* (acetyl-CoA carboxylase beta) was downregulated in BC and positively associated with survival time, which may be a potential target to reduce drug resistance in tumor cells [124]. A previous study reported that the *PDK4* (Pyruvate dehydrogenase kinase 4) gene, involved in regulating glucose metabolism and mitochondrial respiration, is relatively upregulated in BC [125]. Notably, elevated *PDK4* expression has been linked to the promotion of antiestrogen resistance in human BC cells [126]. The gene *UBE2C* (Ubiquitin-conjugating enzyme 2C) exhibits elevated expression in both malignant and benign BC lesions, indicating a potential link between *UBE2C* induction and aberrant cell growth [127]. The gene Polo-like kinase 1 (*PLK1*) belongs to the Polo-like kinases family, which intricately regulates essential biological processes, notably cell cycle control [128]. It governs multiple mitotic phases, including centrosome maturation, spindle formation, chromosome segregation, and cytokinesis. Additionally, it contributes to DNA replication during the S phase, modulating the DNA damage response and ensuring genome stability during replication [129]. Notably, elevated *PLK1* expression is associated with aggressive tumor characteristics in Triple Negative breast cancer (TNBC) [130]. We also verified the role of transcriptional (TFs proteins) and post-transcriptional (miRNAs) regulators of HubGs in BC through the literature review as follows: The TFs protein FOXC1 is related to key pathways in many cancers and may be a novel treatment for these cancers [13,21,131]. FOXC1 has been recommended as a crucial prognostic biomarker with significant functional relevance in BC [132]. The transcription factor GATA2 is overexpressed in human breast carcinomas and promotes BC cell growth [133]. SRF is a potential prognostic biomarker of different types of cancers (such as breast cancer, prostate cancer, and gastric cancer) and may also represent a therapeutic target in the treatment of these cancers [134,135,136]. The hsa-miR-27a-3p miRNA has been identified as a key miRNA in several tumors, including breast [137], ovarian [138], pancreatic [139], and gastric [140] cancer. So, miR-27a-3p may be considered a therapeutic target for BC. In BC cells, down-regulation of miR-16-5p correlates with enhanced migratory and proliferative capabilities, promoting cell cycle advancement and diminishing apoptosis [141]. Conversely, miR-155-5p, a recognized oncogenic microRNA, is frequently upregulated in various malignancies, including BC [142].

We identified some BC-causing Gene Ontology (GO) terms and KEGG pathways through the enrichment analysis of HubGs; those were also supported by some literature reviews. Notably, *PLK1* and *UBE2C* were enriched in “Regulation of Ubiquitin Protein Ligase Activity” and “Mitotic Cell Cycle Phase Transition” BPs. *UBE2C’*s involvement in ubiquitin-mediated proteasome degradation of cell cycle progression in BC was reported by Chow et al. (2014) [127]. Dysregulated E3 ubiquitin ligase functions impact cancer cell behavior, including BC metastasis [114]. *PLK1′*s link to atypical mitosis in BC was demonstrated recently [143]. Another BP, or signal transduction pathway, governs diverse cell functions, including cell division, growth, metabolism, death, and movement [144]. It offers insights into BC progression, presenting therapeutic targets and diagnostic potential [145]. Moreover, dysregulated fatty acid metabolism contributes to malignant transformation in various BC subtypes [146]. Among the identified CC terms, Membrane Raft and Platelet Alpha Granule Membrane are highly associated with BC [147,148]. Many studies have revealed an adverse link between elevated platelet counts and disease-specific survival in various cancers, including BC [149]. The Anaphase-Promoting Complex (APC), a multi-subunit ubiquitin ligase, guides mitotic and G1 progression while contributing to genomic stability [150]. It also plays a pivotal role in cancer through somatic mutations [151]. APC disruption confers resistance to TTK inhibitors in TNBC [152]. Another CC term, extracellular matrix (associated with the HubGs *COL11A1* and *COL10A1*), is involved in the development and progression of cancer and is effective for cancer therapy [153,154]. A variety of chances to find BC druggable targets may be offered by it-mediated signaling pathways [155]. Understanding the intricate links between the ECM and cellular functions has aided in the discovery of disease markers and therapeutic targets [156]. The top-ranked five MFs (Protein Tyrosine Kinase Activator Activity, Protein Kinase Activator Activity, ATP Binding, Transforming Growth Factor Beta Binding, and Ubiquitin Conjugating Enzyme Activity) play a vital role in BC development and proliferation [127,157,158,159,160]. We identified the top five enriched common KEGG pathways (adipocytokine signaling pathway, protein digestion, and absorption, insulin resistance, AMPK signaling pathway, fatty acid biosynthesis) that were also reported by some individual research. Prior reviews have discussed correlations between the Adipocytokine signaling pathway (linked to HubGs *CD36* and *ACACB*) and breast cancer cells, offering novel insights for prevention and treatment [161,162]. Higher insulin and/or C-peptide levels, indicative of insulin resistance, are linked to increased recurrence and mortality risks in early-stage BC, irrespective of diabetes [163]. Adenosine monophosphate-activated protein kinase (AMPK), a crucial metabolic regulator, maintains cellular energy balance and influences diverse physiological and metabolic processes, including glucose and lipid metabolism. Aberrant AMPK signaling contributes to obesity, diabetes, inflammation, and cancer development [164]. AMPK activation can induce p53-dependent apoptosis in BC cells [164,165].

To assess the performance of HubGs on the survival of BC patients, we constructed disease-free survival (DFS) and progression-free survival (PFS) probability curves based on the expressions of HubGs from the TCGA database. Both DFS and PFS probabilities should be lower for the higher expressions of upregulated HubGs compared to their lower expressions. Conversely, these survival probabilities should be higher due to the higher expressions of down-regulated HubGs compared to their lower expressions. From Figure 5A,B, we observed that higher expressions of two upregulated HubGs (*COL11A1* and *CD24*) exhibited lower DFS and PFS probabilities compared to their lower expressions, which satisfied the expected impact on survival. Another three upregulated HubGs (*PLK1*, *UBE2C*, and *COL10A1*) and two downregulated HubGs (*PDK4* and *ACACB*) partially supported the expected impact on survival. For example, the genes *PLK1*, *UBE2C*, and *PDK4* fully satisfied the expected impacts on DFS but partially satisfied the expected impact on PFS up to 120 months. The gene *ACACB* significantly satisfied the expected impact on DFS, and the higher expressions of the up-regulated gene *COL10A1* exhibited the expected lower probabilities in DFS and PFS after 40 and 60 months, respectively, although the difference between the two curves was statistically insignificant. The remaining downregulated gene, *CD36*, did not significantly satisfy the expected outcome. The unsatisfactory results might be due to data errors or other factors that are associated with the survival of patients. From the RF-based prediction model, we found that our prediction model performs well in both test and independent test datasets, with an AUC > 0.96 and an ACC > 0.88 (see Figure S2 and Table S3). The expression analysis of HubGs by Box plots based on the TCGA database in different BC development stages (control, stages-1, stage-2, stage-3, and stage-4) indicated that the proposed HubGs might be utilized as strong diagnostic and prognostic biomarkers at an earlier stage (see Figure 6).

Finally, we recommended our proposed BC-causing five upregulated (*COL11A1*, *COL10A1*, *CD24*, *PLK1*, *UBE2C*) and three down-regulated (*PDK4*, *CD36*, *ACACB*) HubGs-guided top-ranked six ligands/molecules (Suramin, Rifaximin, Telmisartan, Tukysa Tucatinib, Lynparza Olaparib, TG.02) as the candidate drug molecules by molecular docking analysis. It should be mentioned here that both upregulated and down-regulated HubGs were used as drug targets in different studies [166,167,168,169]. Upregulated HubGs-guided drugs inhibit the upregulation of HubGs, while down-regulated HubGs-guided drugs activate the downregulation of HubGs [170]. The molecular docking analysis also showed that our proposed drug molecules significantly bind to the previously suggested BC-causing top-ranked HubGs (*AURKA*, *BUB1*, *FN1*, *TPX2*, *CDC20*, *CCNA2*, *CCNB1*, *BUB1B*, *CDK1* and *TOP2A*)-mediated receptor proteins. We observed that the binding affinity of the top three molecules/ligands (Suramin, Rifaximin, and Telmisartan) was highly significant for all receptors. We verified their resistance power against some cancer-related PTM sites (succinylation, ubiquitination, and phosphorylation) of hub proteins by molecular docking analysis and found their significant binding affinities. The literature review also supported our proposed ligands Suramin [171], Rifaximin [172], Telmisartan [173], Phenylpropan [174], Tukysa Tucatinib [175,176] Lynparza Olaparib [177], and TG.02 [14,178] as the candidate drug molecules for the treatment against BC. Therefore, the findings of this study might be promising and useful resources to wet-lab researchers and clinicians for further investigation to develop an early treatment plan against BC.

## 5. Conclusions

In this study, we identified 8 cDEGs (*COL11A1*, *COL10A1*, *CD36*, *ACACB*, *CD24*, *PLK1*, *UBE2C*, and *PDK4*) as breast cancer (BC)-causing HubGs from 3 microarrays and one scRNA-seq dataset by the protein-protein interaction (PPI) network analysis of 46 cDEGs. The HubGs-set enrichment analysis with GO terms and KEGG pathways disclosed some important biological processes, cellular components, molecular functions, and pathways that are associated with BC progression. We suggested the top-ranked 3 TFs proteins (FOXC1, GATA2, and SRF) and 3 miRNAs (hsa-mir-16-5p, hsa-miR-155-5p, and hsa-mir-27a-3p) as the crucial transcriptional and post-transcriptional regulators of HubGs. The survival probability curves based on the expressions of HubGs in the TCGA database showed that some of the proposed HubGs, including *COL11A1* and *CD24*, might be considered as potential diagnostic and prognostic biomarkers. The random forest (RF)-based BC prediction model also showed good performance in both test and independent test datasets, which indicated its strong prognostic ability. The expression analysis of HubGs based on the independent TCGA database in different BC progression stages (control group, stage-1, stage-2, stage-3, and stage-4) indicated that the proposed HubGs could be considered diagnostic and prognostic biomarkers at an earlier stage. Finally, we recommended HubGs-guided top-ranked six ligands (Suramin, Rifaximin, Telmisartan, Tukysa Tucatinib, Lynparza Olaparib, and TG.02) as the candidate drug molecules for the treatment against BC, in which the top three ligands (Suramin, Rifaximin, and Telmisartan) significantly bound with all receptors. We also verified their resistance power against some cancer-related PTM sites (succinylation, ubiquitination, and phosphorylation) of hub proteins by molecular docking analysis and observed their significant binding abilities. Therefore, the findings of this study might be useful resources to develop a proper treatment plan against BC progression at an earlier stage.

## Figures and Tables

**Table 1:** Selection of HubGs based on three topological measures (A, B, C) through the cytoHubba plugin in Cytoscape.

Degree (A)	Closeness (B)	Betweenness (C)	HubGs (A∪B∪C)
*PLK1* *CD36* *COL10A1* *CD24* *PDK4*	*PLK1* *CD36* *PDK4* *COL10A1* *COL11A1*	*PLK1* *PDK4* *UBE2C* *COL10A1* *ACACB*	*COL11A1*, *COL10A1*, *CD36*, *ACACB*, *PDK4*, *CD24*, *PLK1*, *UBE2C*

**Table 2:** Top five significantly enriched BC-causing GO-terms and KEGG pathways by HubGs.

Term	Count	Adj *p-*Value	Hub Genes
**Biological Process**			
GO:1904666~Regulation of Ubiquitin Protein Ligase Activity	2	0.002	*UBE2C*, *PLK1*
GO:1901990~Regulation of Mitotic Cell Cycle Phase Transition	2	0.018	*UBE2C*, *PLK1*
GO:0043062~Extracellular Structure Organization	2	0.021	*COL11A1*, *COL10A1*
GO:0006631~Fatty Acid Metabolic Process	2	0.023	*CD36*, *ACACB*
GO:0009966~Regulation Of Signal Transduction	2	0.030	*CD36*, *CD24*
**Cellular Component**			
GO:0045121~Membrane Raft	2	0.048	*CD36*, *CD24*
GO:0005788~Endoplasmic Reticulum Lumen	2	0.048	*COL11A1*, *COL10A1*
GO:0031092~Platelet Alpha Granule Membrane	1	0.048	*CD36*
GO:0005680~Anaphase-Promoting Complex	1	0.048	*UBE2C*
GO:0062023~Collagen-Containing Extracellular Matrix	2	0.048	*COL11A1*, *COL10A1*
**Molecular Function**			
GO:0030296~Protein Tyrosine Kinase Activator Activity	2	0.0013	*CD36*, *CD24*
GO:0030295~Protein Kinase Activator Activity	1	0.006	*CD36*, *CD24*
GO:0005524~ATP Binding	2	0.029	*PLK1*, *PDK4*
GO:0050431~Transforming Growth Factor Beta Binding	1	0.030	*CD36*
GO:0061631~Ubiquitin Conjugating Enzyme Activity	1	0.030	*UBE2C*
**KEGG Pathways**			
Adipocytokine signaling pathway	2	0.004	*CD36*, *ACACB*
Protein digestion and absorption	2	0.004	*COL11A1*, *COL10A1*
Insulin resistance	2	0.004	*CD36*, *ACACB*
AMPK signaling pathway	2	0.004	*CD36*, *ACACB*
Fatty acid biosynthesis	1	0.024	*ACACB*

**Table 3:** Binding affinities of the top-three proposed ligands with the BC-causing three types of PTM sites (Succinylation, phosphorylation, and ubiquitination) of the top-ranked three hub proteins.

Top Three Ligands	Succinylated Sites of ACACB Protein										
	K83	K158	K228	K305	K435	K722	K1264	K1449	K1469	K1473	K2246
Suramin	−9	−13.1	−8.2	−8.1	−12.1	−8.3	−8.2	−8.4	−7.8	−8.7	−6.7
Rifaximin	−9.2	−8.5	−12.1	−9	−8.7	−7.1	−7.3	−6.9	−6.8	−8.8	−8.2
Telmisartan	−9	−7.1	−8.2	−6.9	−7.1	−7.3	−9.5	−8.8	−8.3	−7.2	−8.1
Top three ligands	**Succinylated sites of PDK4 protein**				**Succinylated sites of CD36 protein**						
	K142				K213	K218	K223	K231	K286	K403	K406
Suramin	−9.5				−7.5	−8.2	−7.5	−8.1	−9.5	−9	−7.1
Rifaximin	−7.6				−8.2	−10.2	−8.4	−8.3	−7.2	−6.9	−7.7
Telmisartan	−5.3				−7.5	−7.3	−8.2	−9	−9.5	−9.1	−8.5
Top three ligands	**Phosphorylated sites of ACACB protein**										
	S302	S350	T70	S72	S175	S195	S246	S469	S1360	T2025	
Suramin	−10	−7.2	−7.1	−8.5	−12.1	−6.2	−5.6	−9.1	−8.2	−7.5	
Rifaximin	−8.3	−5.3	−8.1	−9.1	−8.7	−12.1	−8	−8.2	−7.6	−9.1	
Telmisartan	−8	−10	−7	−7.1	−8.8	−7.9	−7.8	−7.3	−9	−11.1	
Top three ligands	**Phosphorylated sites of PDK4 protein**							**Phosphorylated sites of CD36 protein**			
	S10	S13	S33	S106	S222	T316	S390	S302	S350		
Suramin	−8.1	−7.6	−8.8	−7.7	−8.3	−8.9	−7.2	−7.3	−9.5		
Rifaximin	−8.8	−8.2	−7.5	−8.1	−8.1	−9.1	−6.8	−11.2	−8.7		
Telmisartan	−7.2	−8.7	−7.9	−12.1	−7	−7.1	−8.4	−9	−7.9		
Top three ligands	**Uubiquitinated sites of ACACB protein**										
	S302	S350	T70	S72	S175	S195	S246	S469	S1360	T2025	
Suramin	−9.2	−7.8	−8.1	−8	−9.2	−7.4	−8.1	−8.1	−9	−7	
Rifaximin	−7.1	−8.5	−8.9	−7.1	−8.5	−6.7	−8.8	−7.9	−8.7	−8.4	
Telmisartan	−8.3	−8.9	−8.1	−7.5	−8.5	−8.1	−5.6	−10	−7.2	−6.9	
Top three ligands	**Ubiquitination sites of PDK4 protein**							**Ubiquitinated sites of CD36 protein**			
	S10	S13	S33	S106	S222	T316	S390	S302	S350		
Suramin	−7.8	−7.3	−10.2	−9.1	−6.8	−8.1	−9.1	−8.8	−8.2		
Rifaximin	−7	−9.5	−8	−5.6	−8.8	−9.1	−8.7	−8.5	−7.3		
Telmisartan	−7.3	−7.9	−7.8	−7.7	−8.9	−7.1	−8.8	S302	S350		

**Table 4:** The 3D view of the top-order three drugs is shown in column 3, and protein and ligand interactions are shown in the 4th column.

Protein and Ligand	Binding Affinity (kCal/mol)	The 3D View of Complex	Protein and Ligand Interaction	Interacting Amino Acids	
				Hydrogen Bond	Hydrophobic Interactions
PDK4 and Rifaximin	−11	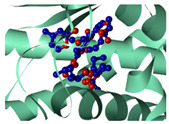	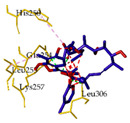	LYS257, UNL1, LEU306, GLU254	LEU255, HIS250
ACACB and Suramin	−9	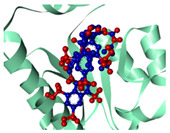	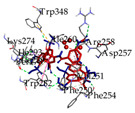	ASP257, ARG258, UNL1, LYS274, SER278, TRP282, TRP348, ARG258, PHE254, ILE279	TRP282, UNL1, ILE260, ILE293, VAL251, ILE279
CD36 and Telmisartan	−8.6	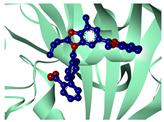	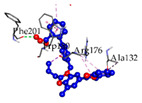	UNL1, PHE201	UNL1, TRP180, ALA120, ARG176, ALA132, ARG176

## Data Availability

The gene-expression profiles datasets were downloaded from the GEO platform in the NCBI database with accession numbers GSE26910, GSE42568, GSE65194, GSE45827, and GSE54002 weblinks: https://www.ncbi.nlm.nih.gov/geo/query/acc.cgi?acc=GSE26910, https://www.ncbi.nlm.nih.gov/geo/query/acc.cgi?acc=GSE42568, https://www.ncbi.nlm.nih.gov/geo/query/acc.cgi?acc=GSE65194, https://www.ncbi.nlm.nih.gov/geo/query/acc.cgi?acc=GSE45827, and https://www.ncbi.nlm.nih.gov/geo/query/acc.cgi?acc=GSE54002, respectively (accessed on 12 January 2023). Also, one scRNA-seq dataset (GSE235168) was downloaded from the NCBI database with the weblink https://www.ncbi.nlm.nih.gov/geo/query/acc.cgi?acc=GSE235168 (accessed on 22 August 2023). The other relevant data are given in the Supplementary Tables.

## Supplementary Materials

The following supporting information can be downloaded at: https://www.mdpi.com/1648-9144/59/10/1705/s1. Figure S1: Differential expression pattern analysis of HubGs with Boxplots by TCGA and GTEx data Figure S2: The ROC curves for the Random Forest (RF)-based breast cancer prediction model Red and blue indicate the prediction performance of the test and independent test datasets. Table S1: Breast cancer (BC)-causing hub-DEGs (HubGs) sets, including datasets and analysis methods collected from 59 articles by the literature review [1,14,23,24,25,26,27,28,29,30,31,32,33,34,35,36,37,38,39,40,41,42,43,44,45,46,47,48,49,50,51,52,53,54,55,56,57,58,59,60,61,62,63,64,65,66,67,68,69,70,71,72,73,74,75,76,77,78,79] Table S2: Drug lists collected from different sources [11,12,90,179] Table S3: Test performance scores of the RF-based prediction model with the cutoff at FPR ≤ 15%. Table S4: Binding affinity scores for the proposed top-ranked six ligands with the succinylated PTM sites of hub-proteins. Table S5: Binding affinity scores for the proposed top-ranked six ligands with the phosphorylated PTM sites of hub-proteins. Table S6: Binding affinity scores for the proposed top-ranked six ligands with the ubiquitinated PTM sites of hub-proteins.
