# Supplementary material for: Robust Identification of Differential Gene Expression Patterns from Multiple Transcriptomics Datasets for Early Diagnosis, Prognosis, and Therapies for Breast Cancer
Source: Medicina (Kaunas). 2023 Sep 24;59(10):1705. doi: 10.3390/medicina59101705 (PMC10608013; doi:10.3390/medicina59101705)
Supplement: Supplementary file 1 [file medicina-59-01705-s001.zip › medicina-2545125-supplementary.pdf]

Robust Identification of Differential Gene Expression Patterns from Multiple Transcriptomics Datasets for Early Diagnosis, Prognosis, and Therapies for Breast Cancer

Khanis Farhana Tuly [1], Md. Bayazid Hossen [1], Md. Ariful Islam [1], Md. Kaderi Kibria [1,2], Md. Shahin Alam [1], Md. Harun-Or-Roshid [1], Anjuman Ara Begum [1], Md. Sohel Hasan [3], Rashidul Alam Mahumud [4] and Md. Nurul Haque Mollah [1*]

(Supplementary file)

Supplementary Figures/ Tables legends	Pages
**Supplementary Figure:**	
**Figure S1.** Differential expression pattern analysis of HubGs with Boxplots by TCGA and GTEx data	2
**Figure S2.** The ROC curves for the Random Forest (RF) based breast cancer prediction model. Red and blue indicates the prediction performance of the test and independent test datasets.	3
**Supplementary Table:**	
**Table S1:** Breast cancer (BC) causing hub genes (HubGs) sets including datasets and analysis methods collected from 60 articles by the literature review.	4-8
**Table S2:** Drug lists collected from different sources	8-9
**Table S3.** Test performance scores of the RF-based prediction model with the cutoff at FPR ≤ 15%	10
**Table S4.** Binding affinity scores for the proposed top-ranked six ligands with the **succinylated** PTM sites of hub-proteins	10-12
**Table S5**. Binding affinity scores for the proposed top-ranked six ligands with the phosphorylated PTM sites of hub-proteins	12-14
**Table S6**. Binding affinity scores for the proposed top-ranked six ligands with the **ubiquitina**ted PTM sites of hub-proteins	15-17

Supplementary Figures

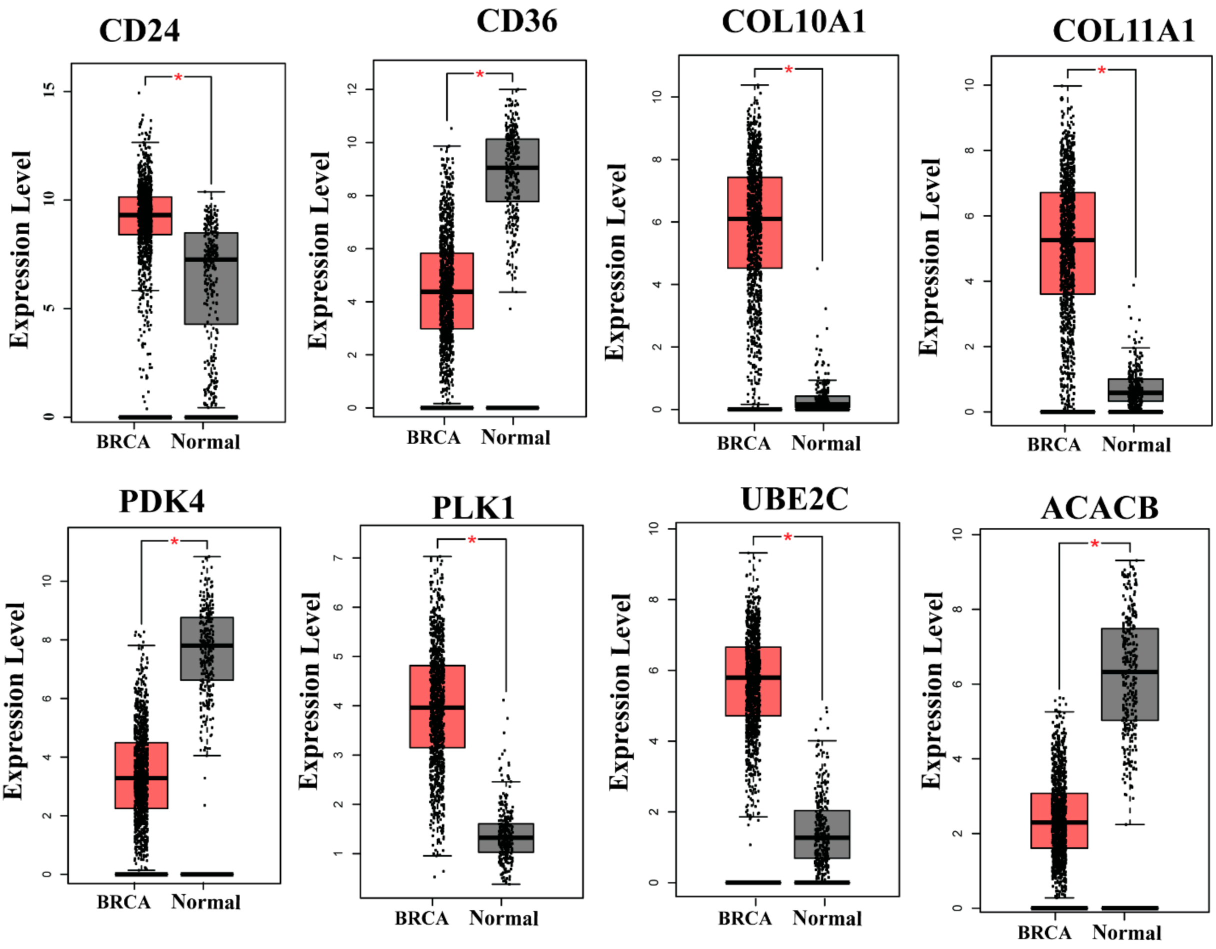

Figure S1. Differential expression pattern analysis of HubGs with Boxplots by TCGA and GTEx data

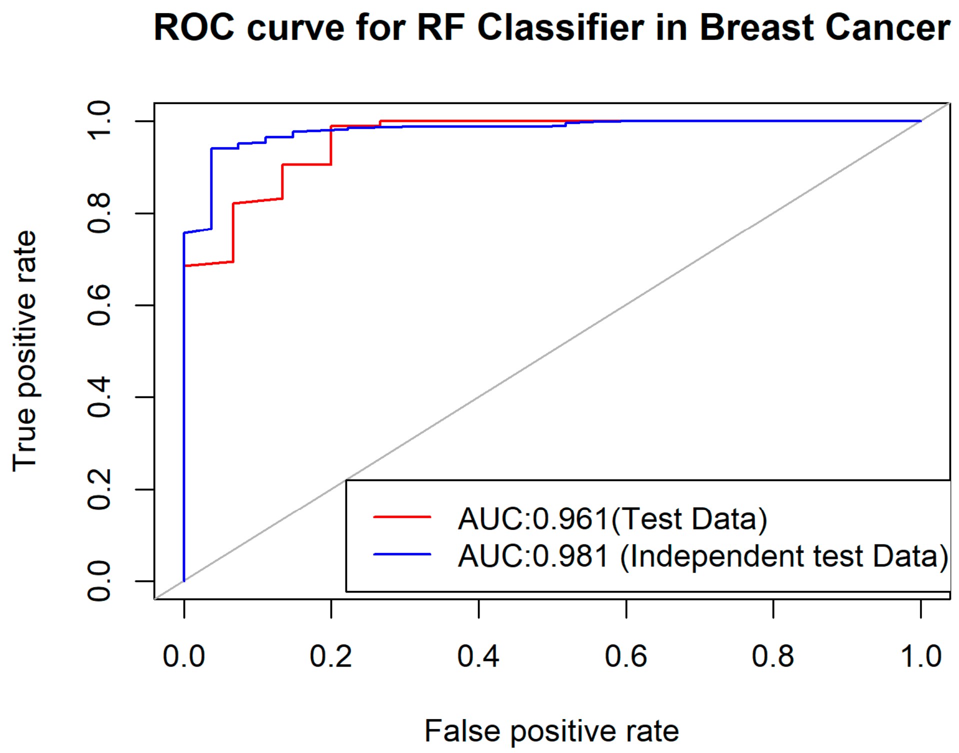

Figure S2. The ROC curves for the Random Forest (RF)- based breast cancer prediction model. Red and blue indicates the prediction performance of the test and independent test datasets.

Supplementary Tables

Table S1: Breast cancer (BC) causing hub genes (HubGs) sets including datasets and analysis methods collected from 59 articles by the literature review.

Articles	Hub-DEGs (HubGs) sets	Datasets	Methods for detection of DEGs
Wu et al. 2020[37]	BUB1B, CDC45, MAD2L1, PLK1, CDC20, AURKA	GSE23720, GSE45581	LIMMA
Wang et al. 2020 [38]	CCNE1, CHEK1, PLK1, CENPN, DSCC1, FAM64A, UBE2T, UBE2C	TCGA dataset	LIMMA
Wu et al. 2020 [39]	FOXA1, GATA3, ESR1, PGR, CCND1, AR, TFF1, GREB1, NRIP1, KRT18	GSE22093, GSE23988	LIMMA
Chen et al. 2020 [40]	RPL4, HSP90AA1, SRC, ESR1, PPP2CA, HSPA8, ACTB	GSE86945, GSE10208 and 8GSE86946	LIMMA
Zhou et al. 2020 [41]	CXCL8, CD44, BMP7, MMP9	GSE74146	LIMMA
Yang et al. 2019 [1]	TPX2, CCNB2, BUB1, CENPE, KIF2C, NDC80, TOP2A, MELK, KIF20A, CKS2	GSE25066, GSE21422	LIMMA
Liu et al. 2021 [42]	ITGB1, FERMT2, ITGA5, CEMIP, HGF, MCAM, TGFBR1, F2RL2	GSE12276, GSE20685, and GSE16446	LIMMA
Chuan et al. 2020 [43]	CXCR4, CXCL10	GSE65194, GSE53752, GSE45827 and GSE38959	LIMMA
Jin et al. 2018 [44]	CCNB1, AURKA, BIRC5, BUB1B, ZWINT, CCNB2, CDK1, CDKN3, CENPF, PTTG1, TOP2A, CDC20, TPX2, UBE2C, PRC1	GSE10810, GSE65194 and TCGA dataset	LIMMA
Tian et al. 2018 [45]	GGTA1P, CRABP2, SYNPO2, NAP1L2, MKI67, COL4A6, MAGI2-AS3	GSE60689, GSE64790	LIMMA
Shi et al. 2020 [46]	BUB1B, CCNB2, AURKA, CDK1, CDT1, KIF20A, KIF2C, HJURP, KIF4A, MELK, UBE2C, TPX2	GSE42568, GSE102484, GSE20685, GSE21653 and TCGA dataset	LIMMA

Li et al. 2018 [47]	PLK1, AURKA, BIRC5, BUB1, CCNB1, BUB1B, CDK1, MAD2L1, NDC80, KIF11	GSE45827, GSE38959, and GSE65194	LIMMA
Liu et al. 2020 [48]	CDC20, CCNA2, CDK1, CCNB1, CCNB2, BUB1B, CDCA8, BUB1, KIF11, TOP2A	GSE24124, GSE32641, GSE36295, GSE42568, GSE53752, GSE70947, GSE109169)	LIMMA
Zhang et al. 2020 [49]	HSPD1, HSPH1, TXN, ATIC	GSE26459	LIMMA
Wei et al. 2021 [50]	CDK1, TOP2A, CCNB1, MKI67, TTK, BUB1, PLK1, CCNA2	GSE76250	LIMMA
Dong et al. 2018 [51]	AR, SOX8, RAB30, C9orf152, NRK	GSE76275	LIMMA
Wu et al. 2012 [52]	MCM3, BCL2, MCM7, TGFB2	GSE11352	LIMMA
He et al. 2021 [53]	TGFBR3, C1QB, CEP55, SPP1, IFI6, KIAA0101, PBK, DCN, FZD7, KRT5, HIST1H2BO, SPAG5	GSE26338	LIMMA
Zhu et al. 2020 [54]	CXCL12, CXCL12, FOS	GSE59733	LIMMA
Li et al. 2021 [55]	CD3G, CD3D, CD3E, GRAP2, FYN, ITK	GSE43358, GSE27447, GSE39004, and GSE45827	LIMMA
Xiao et al. 2021 [56]	NCAPG, CCNB1, MCM4, RRM2	GSE38959 ,GSE65212	LIMMA
Lu et al. 2019 [57]	ACACB, PHLPP1, UBC, TGFB1, ACTB	GSE43837	LIMMA
Wang et al. 2019 [58]	MELK, CDK1, TOP2A, CCNB1, KIF11, CCNA2	GSE29431, GSE42568, GSE21422, and GSE61304	LIMMA
Yan et al. 2021 [59]	FN1, DDR2, EFNA3, RHOQ, NRP1	GSE128610 and GSE72319	LIMMA
Peng et al. 2020 [60]	TP53, SMURF1, BTRC	GSE101123	LIMMA
Lin et al. 2020 [61]	TOP2A, CCNB1, RAC1, KIF20A, NUSAP1, RRM2, ASPM, BIRC5, CEP55, BUB1B	GSE29431, GSE45827, and GSE65194	LIMMA
Zhou and Yang 2020 [62]	IL1β, PTGS2, CXCL8	GSE3365 and GSE27562	LIMMA

Lv et al. 2020 [63]	IL6, PTPRC, SELL, SPN, CD40	GSE5847, GSE22597, GSE23720, and GSE45581	LIMMA
Bai et al. 2020 [64]	STAT3, STAT1, STAT6, STAT2, XBP1, BCL2L1, ESCO2, CYB5D2	GSE21748	LIMMA
Hao et al. 2020 [11]	RRM2, CDC20, CCNB2, BUB1B, CDK1, CCNA2	GSE42568, GSE61304, GSE374, GSE21422, and GSE65194	LIMMA
Wang et al. 2019 [65]	SIDT1, GPR160, ANKRD30A, CA12	GSE76275	LIMMA
Pei et al. 2020 [66]	CCNB2, TPX2, EXO1, CENPA, SKA1, FOXM1, PLK1, SGO1, RAD54L, KIF4A, CDC20	TCGA dataset	LIMMA
Tang et al. 2019 [67]	VCAM1, ANLN, BUB1, SKA3, JUN, NUSAP1, CKAP2L, TTK, FOS, STAT5A	GSE100534 and GSE52604	LIMMA
Qi et al. 2019 [68]	TOP2A, MAD2L1, PIK3R1, FEN1, EPRS, EXO1, BCL2, MCM4, PTTG1, PSMD14, CDKN3, RRM2, H2AFZ, CCNE2, FGF2, EGFR	GSE20713, GSE54002, GSE65194, and GSE32393	LIMMA
Wang et al. 2018 [69]	JUN, ATF3, STAT1, FN1, FOS, COL1A1	GSE26910 and GSE10797	LIMMA
Fang and Zhang 2017 [70]	RPS14, RPS9, RPL11, RPL10A	GSE10797	LIMMA
Lou et al. 2018 [71]	PRDM10, MAPK1	GSE73736	LIMMA
Bao et al. 2019 [72]	BCL11A, FAM171A1, FOXC1, RGMA	TCGA dataset	LIMMA
Zhong et al. 2020 [73]	CT83, FABP7, ART3, TTYH1	GSE76275, GSE36693, GSE27447, GSE3744	LIMMA
Wang et al. 2018 [74]	TOP2A, BIRC5, NDC80, CDK1, CCNB1	GSE5764, GSE21422, GSE22035, GSE3744, and GSE26910	LIMMA
Dashti et al. 2020 [75]	CCNA2, MAD2L1, RAD51-AS1, LINC01089	GSE65194 and GSE45827	LIMMA
Liu et al. 2015 [76]	FN1, FOS, IL6	GSE26910	LIMMA
Bao et al. 2020 [77]	AURKB, GINS2, UHRF1, POLE2, SPC24, E2F2, MCM10	GSE38376, GSE16179, , and GSE51889	LIMMA

Zhang et al. 2019 [78]	UBA5, CREB1, ARF3, SIAH1, KLHL3, TRIM69, HECTD1, MMP9, MEX3C, UBE2Q2, FBXO22, ASB6, EIF4A3, PXN	GSE31192 and GSE53031	LIMMA
Zheng et al. 2017 [79]	TP53INP1, SPI1, CAPG, LEF1, PBX3, PLAGL1, EGFR, TCF7L2, SYK	GSE8977	LIMMA
Alam et al. 2022 [14]	AKR1C1, IRF9, SLCO2A1, NT5E, NQO1, FN1, OAS1, OAS3, ATF6B, HPGD, ANGPT1, BCL11A, TP53INP1	GSE53566	LIMMA
Alam et al. 2022 [24]	CCNA2, RFC4, CCNB1 , BUB1, ASPM, TTK, CENPF	GSE45827, GSE42568, GSE139038, GSE62931, and GSE54002,	LIMMA & SAM
Hong et al. 2020 [35]	CCNB2, CDC20, PTTG1, CCNB1, BUB1B, CCNE2, TTK	GSE42568, GSE45827 and GSE54002	LIMMA & EdgeR
Zhao et al. 2019 [36]	KRAS, CDH2, VEGFA, ZEB2, NTRK2, TWIST1	GSE101123 and TCGA dataset	LIMMA, EdgeR
Li et al. 2020 [29]	TTK, CCNE1, EXO1	TCGA dataset	EdgeR
Lv et al. 2019 [30]	CDC20, CCNA2, CCNB2, TTK, CENPF, BUB1, CENPA, CENPE	TCGA dataset	EdgeR
Yuan et al. 2019 [31]	FN1, EGFR, PTPRC, JAK3, TUBB3	GSE139274	EdgeR
Cai et al. 2019 [27]	KIF4A, TPX2, CDCA8, CCNA2, BUB1B	GSE102484	WGCNA
Fu et al. 2019 [28]	KIF18B, CASC5, CKAP2L, KIF23, SKA1, CDCA5, MCM6, FAM83D, GINS1	GSE102484	WGCNA
He et al. 2015 [32]	MYEOV2, DUSP1, UQCRQ	GSE27447, GSE18864	heatmap.2
Takeshita et al. 2020 [25]	CTNNB1, TP53, EGFR, ERBB2, HSPB1	GSE32603 and GSE87455	t-test
Wang et al. 2018 [26]	MAPK14, NOTCH1	GSE71142	t-test
Zeng et al. 2018 [33]	FGF2, GSK3B, RAC1, ERBB2, PIK3R1, PXN, RAC2, HSP90AA1	GSE22358	GENE-E
Qin and Chen 2016	FSTL3, TUBA1C, DUSP8, KLF6,	E-GEOD-50811	SAM

[23]	EIF3B, KIF20A, PTPRK, UBR2, ZSCAN20, DEPDC1, AURKA, UNG		
Amjad et al. 2020 [34]	FOS, SMC4, FN1, KIF11, RACGAP1, JUN	GSE129551, GSE124647, and GSE124646	The BRBArrayTools (v4.6.0)
**Common hub genes with at least 5 articles:**	AURKA, BUB1, FN1, TPX2, CDC20, CCNA2, CCNB2, CCNB1, BUB1B, CDK1, TOP2A		

Table S2: Drug lists collected from different sources

Drug Lists	Sources
Gallium Notrate, Cladribine, Hydroxyurea, Motexafin Gadolinium, Gemcitabine, Clofarabine, Fludarabine Phosphate, Dinaciclib, Triapine, Fludarabine, AT-7519, Alvocidib, AZD-5438, Roniciclib, TG-02, Genistein, Cordycepin, Suramin, seliciclib, RG-547, CHEMBL1236539	Hao et al. 2020 [11]
Progesterone, Levonorgestrel , Estropipate, Tamoxifen, Estradiol, Danazol, Diethylstilbestrol, Desogestrel, Mestranol, Clomifene, Polyestradiol Phosphate, Fulvestrant, Norgestimate, Medroxyprogesterone, Estrone, Dienestrol, Naloxone, Tibolone, Quinestrol, Ethynodiol Diacetate, Fluoxymesterone, Estramustine, Promestriene, Estriol, Toremifene, Lasofoxifene, Etonogestrel, Trilostane, Ospemifene, Norgestrel, Allylestrenol, Raloxifene, Megestrol, Conjugated Estrogen, Bazedoxifene, Ethinyl Estradiol, Chlorotrianisene	Dong et al. [12]
15deltaProstaglandin, Acetovanillone, ACMC20mvek, Alprostadil, Anandamide, Benfotiamine, Benserazide, Bezafibrate, BISPHENOL_2286, BisphenolA, cacodylicAcid, candesartan, choline, deferoxamine, deoxythymidine_5726, dexamethasone, DICHLOROANILINE_7257, Dinoprostone, Electrocorundum, Ellipticine, Fenofibrate, Forskolin, Geldanamycin, GW9662, hydroxybenzoate_7184, IBMX, Ibuprofen, indole3aceticAcid, indomethacin, ligand, losartan, L_sorbose, Malondialdehyde, Melatonin, Metformin, Mifepristone, MK, oleicAcid, Oxazolone, Oxozinc, Oxygen, palmiticAcid, PerfluoroheptanoicAcid, PerfluorononanoicAcid, PerfluorooctanesulfonicAcid, phenylpropan_5155877, phenytoin, Pioglitazone, RetinoicAcid, Rifaximin, Rofecoxib, Rosiglitazone, salicylicAcid, Silica, Tamoxifen, Tanespimycin, TauroursodeoxycholicAcid, Telmisartan, Tetradioxin, Thioctic_864, Triclosan, Triflumizole, Troglitazone, Valsartan, yc1	Selected by the HubGs-set enrichment analysis with the drugs molecules of DSigDB database [90]

5-FU, Abraxane, AdoTrastuzumabEmtansine, AfinitorDisperzEverolimus, AfinitorEverolimus, Aredia_PamidronateDisodium, ArimidexAnastrozole, LynparzaOlaparib, MegestrolAcetate, AromasinExemestane, EllenceEpirubicinHydrochloride, EribulinMesylate, FarestonToremifene, FaslodexFulvestrant, FemaraLetrozole, Talzenna, Fulvestrant, Gemzar_GemcitabineHydrochloride, GoserelinAcetate, HalavenEribulinMesylate, IbrancePalbociclib, IxempraIxabepilone, Kadcyla, KisqaliRibociclib, MethotrexateSodium, NeratinibMaleate, NerlynxNeratinibMaleate, Tykerb, PamidronateDisodium, PiqrayAlpelisib, SoltamoxTamoxifen, Talazoparib_Tosylate, Zoladex, TamoxifenCitrate, TaxotereDocetaxel, Tepadina-Thiotepa, Trexall, TukysaTucatinib, VerzenioAbemaciclib, VinblastineSulfate, Xeloda,	FDA approved Drugs for Breast cancer [179]
**Combined list of 142 drugs by taking union of all drug lists mentioned above, is given below**	
15deltaProstaglandin, 5_FU, Abraxane, Acetovanillone, ACMC20mvek, AdoTrastuzumabEmtansine, AfinitorDisperzEverolimus, AfinitorEverolimus, Allylestrenol, Alprostadil, ALVOCIDIB, Anandamide, Aredia_PamidronateDisodium, ArimidexAnastrozole, AromasinExemestane, AT_7519, AZD_5438, Bazedoxifene, Benfotiamine, Bezafibrate, BISPHENOL_2286, BisphenolA, Candesartan, CHEMBL1236539, Chlorotrianisene, Choline, CLADRIBINE, CLOFARABINE, Clomifene, conjugated estrogen, CORDYCEPIN, Danazol, Deferoxamine, deoxythymidine_5726, desogestrel, dexamethasone, diacetate, DICHLOROANILINE_7257, Dienestrol, Diethylstilbestrol, DINACICLIB, Dinoprostone, EllenceEpirubicinHydrochloride, Ellipticine, EribulinMesylate, Estradiol, Estramustine, Estriol, Estrone, Estropipate, ethinyl estradiol, ethynodiol, etonogestrel, FarestonToremifene, FaslodexFulvestrant, FemaraLetrozole, Fenofibrate, FLUDARABINE, FLUDARABINE PHOSPHATE, Fluoxymesterone, Forskolin, Fulvestrant, Geldanamycin, GEMCITABINE, Gemzar_GemcitabineHydrochloride, GENISTEIN, GoserelinAcetate, GW9662, HalavenEribulinMesylate, hydroxybenzoate_7184, HYDROXYUREA, IBMX, IbrancePalbociclib, Ibuprofen, indole3aceticAcid, indomethacin, IxempraIxabepilone, Kadcyla, KisqaliRibociclib, Lasofoxifene, Levonorgestrel, Ligand, Losartan, L_sorbose, LynparzaOlaparib, Malondialdehyde, Medroxyprogesterone, MegestrolAcetate, Megestrol, Melatonin, Mestranol, Metformin, MethotrexateSodium, Mifepristone, MK, Naloxone, NeratinibMaleate, NerlynxNeratinibMaleate, Norgestimate, oleicAcid, ospemifene, Oxazolone, Oxygen, palmiticAcid, PamidronateDisodium, PerfluoroheptanoicAcid, phenylpropan_5155877, phenytoin, Pioglitazone, PiqrayAlpelisib, polyestradiol phosphate, progesterone, promestriene, quinestrol, raloxifene, RetinoicAcid, RG_547, Rifaximin, Rofecoxib, Roniciclib, salicylicAcid, SELICICLIB, Silica, SURAMIN, Talazoparib_Tosylate, Talzenna, TamoxifenCitrate, Tamoxifen, Tanespimycin, TauroursodeoxycholicAcid, TaxotereDocetaxel, Telmisartan, Tepadina_Thiotepa, Tetradioxin, TG_02, Thioctic_864, Tibolone, Toremifene, Trexall, TRIAPINE, Triclosan, Triflumizole, Trilostane, Troglitazone, TukysaTucatinib, Tykerb, Valsartan, VerzenioAbemaciclib, VinblastineSulfate, Xeloda, yc1, Zoladex	

Table S3. Test performance scores of the RF-based prediction model with the cutoff at FPR ≤ 15%

	Test Dataset	Independent Test Dataset
Area under the ROC Curve (AUC)	0.961	0.981
Accuracy (ACC)	0.881	0.906
Sensitivity or true positive rate (TPR)	0.867	0.962
Specificity or true negative rate (TNR)	0.852	0.850
False negative rate (FNR)	0.222	0.037
False positive rate (FPR)	0.147	0.149
False discovery rate (FDR)	0.020	0.134

Table S4. Binding affinity scores for the proposed top-ranked six ligands with the **succinylated** PTM sites of hub-proteins

**Top six ligands**	**Succinylated sites of ACACB protein**										
	K83	K158	K228	K305	K435	K722	K1264	K1449	K1469	K1473	K2246	
Suramin	-9	-13.1	-8.2	-8.1	-12.1	-8.3	-8.2	-8.4	-7.8	-8.7	-6.7	
Rifaximin	-9.2	-8.5	-12.1	-9	-8.7	-7.1	-7.3	-6.9	-6.8	-8.8	-8.2	
Telmisartan	-9	-7.1	-8.2	-6.9	-7.1	-7.3	-9.5	-8.8	-8.3	-7.2	-8.1	
Tukysa-Tucatinib	-8.9	-7.2	-8.3	-8.8	-7.7	-9.5	-7.2	-8.9	-9	-6.6	-7.6	
Lynparza-Olaparib	-12.1	-13.1	-8.2	-8.9	-8.5	-7.2	-8.4	-7.3	-9.2	-8.5	-8.4	
TG.02	-8.1	-8.8	-8	-8.4	-7.1	-8.4	-8.9	-6.9	-12.1	-5.6	-7.9	

Top six ligands	Succinylated sites of CD36 protein											
	K213	K218	K223	K231	K286	K403	K406					
Suramin	-7.5	-8.2	-7.5	-8.1	-9.5	-9	-7.1					
Rifaximin	-8.2	-10.2	-8.4	-8.3	-7.2	-6.9	-7.7					
Telmisartan	-7.5	-7.3	-8.2	-9	-9.5	-9.1	-8.5					
Tukysa-Tucatinib	-8.2	-9	-8.9	-7.6	-7.6	-7.5	-7.1					
Lynparza-Olaparib	-7	-8.8	-8.4	-9	-5.3	-7.1	-7.1					
TG.02	-8.9	-9	-6.6	-6.9	-7.1	-8.8	-8.4					
**Top six ligands**	**Succinylated sites of COL10A1 protein**											
	K116	K126	K400									
Suramin	-8.1	-9.1	-6.8									
Rifaximin	-8.3	-8.8	-7.7									
Telmisartan	-9	-6.6	-7.6									
Tukysa-Tucatinib	-8.8	-8.2	-7.5									
Lynparza-Olaparib	-7.8	-8.7	-6.7									
TG.02	-9.2	-8.5	-8.4									
**Top six ligands**	**Succinylated sites of COL11A1 protein**											
	K163	K173	K183	K387	K789	K795	K875	K1019	K1182	K1443		
Suramin	-7.2	-8.4	-7.3	-9.2	-8.2	-7.5	-8.9	-6.8	-6.9	-8.5		
Rifaximin	-8.1		-8.7	-7.1	-7.3	-8.7	-8.4	-8.5	-9.1	-7.1		
Telmisartan	-9	-7.2	-8.3	-7.6	-7.5	-8.5	-7.5	-7.5	-7.5	-7.2		
Tukysa-Tucatinib	-6.9	-8.5	-8.8	-8	-6.8	-7.3	-8.2	-8.4	-7.1	-13.1		
Lynparza-Olaparib	-8.1	-12.1	-9	-7.1	-8.2	-9.5	-8.9	-6.8	-8.8	-8.8		

TG.02	-7.3	-8.2	-9.2	-9.1	-7.1	-7.6	-8.2	-8.5	-8.1	-8.5		
**Top six ligands**	**Succinylated sites of PDK4 protein**											
	K142											
Suramin	-9.5											
Rifaximin	-7.6											
Telmisartan	-5.3											
Tukysa-Tucatinib	-7.7											
Lynparza-Olaparib	-8.5											
TG.02	-7.1											

Table S5. Binding affinity scores for the proposed top-ranked six ligands with the phosphorylated PTM sites of hub-proteins

**Top six ligands**	**Phosphorylated sites of ACACB protein**											
	S302	S350	T70	S72	S175	S195	S246	S469	S1360	T2025		
Suramin	-10	-7.2	-7.1	-8.5	-12.1	-6.2	-5.6	-9.1	-8.2	-7.5		
Rifaximin	-8.3	-5.3	-8.1	-9.1	-8.7	-12.1	-8	-8.2	-7.6	-9.1		
Telmisartan	-8	-10	-7	-7.1	-8.8	-7.9	-7.8	-7.3	-9	-11.1		
Tukysa-Tucatinib	-9	-9.2	-5.3	-8.4	-7.7	-9.2	-7	-9.5	-6.8	-8.1		
Lynparza-Olaparib	-10.2	-9.1	-6.8	-9.5	-8	-8.5	-7.9	-7.2	-8.4	-8.7		
TG.02	-8.1	-7.6	-8.8	-7.7	-8.3	-8.9	-7.2	-8.4	-6.6	-7.4		
**Top six**	**Phosphorylated sites of CD36 protein**											

**ligands**	S302	S350										
Suramin	-7.3	-9.5										
Rifaximin	-11.2	-8.7										
Telmisartan	-9	-7.9										
Tukysa-Tucatinib	-8.2	-9.5										
Lynparza-Olaparib	-6.8	-8.1										
TG.02	-5.5	-6.8										
**Top six ligands**	**Phosphorylated sites of COL10A1 protein**											
	T61	S87	S381	S384	S471	S533	T547					
Suramin	-6.2	-11.2	-9.5	-6.8	-8.1	-8	-8.8					
Rifaximin	-9.3	-8.2	-7.2	-7.9	-12.1	-7.6	-7.2					
Telmisartan	-7.9	-7.5	-8.7	-8.3	-9.5	-7.4	-6.6					
Tukysa-Tucatinib	-7.1	-8.5	-12.1	-8.3	-7.9	-8.2	-8.5					
Lynparza-Olaparib	-6.8	-9.5	-8.7	-7.1	-9.2	-12.1	-5.6					
TG.02	-8.4	-8.3	-8.8	-7.4	-8.3	-9	-8.5					
**Top six ligands**	**Phosphorylated sites of COL11A1 protein**											
	S50	S134	S240	T358	S370	T659	S1058	T1067	S1481	T1558	S1737	
Suramin	-5.6	-7.1	-8.2	-7.5	-7.7	-8.4	-8.7	-7.9	-6.7	-7.1	-8.2	
Rifaximin	-8	-8.4	-7.7	-9.2	-7	-9.5	-6.8	-8.1	-8.4	-7.7	-9.2	
Telmisartan	-8.1	-7.5	-8.5	-8.8	-8	-6.8	-8.9	-7.6	-7.5	-8.5	-8.8	
Tukysa-Tucatinib	-7	-8.8	-8.8	-7.7	-8.3	-8.9	-7.2	-8.4	-6.6	-7.4	-7.7	
Lynparza-Olaparib	-7.9	-7.1	-8.9	-7.1	-8.5	-12.1	-8.3	-7.9	-8.2	-8.5	-7.1	
TG.02	-7.2	-10	-7.2	-7.1	-8.5	-12.1	-6.2	-5.6	-9.1	-8.2	-7.5	
**Top six**	**Phosphorylated sites of PDK4 protein**											

ligands	S10	S13	S33	S106	S222	T316	S390					
Suramin	-8.1	-7.6	-8.8	-7.7	-8.3	-8.9	-7.2					
Rifaximin	-8.8	-8.2	-7.5	-8.1	-8.1	-9.1	-6.8					
Telmisartan	-7.2	-8.7	-7.9	-12.1	-7	-7.1	-8.4					
Tukysa-Tucatinib	-6.6	-8.8	-8	-8.4	-5.3	-8.4	-6.6					
Lynparza-Olaparib	-7.8	-8.7	-6.7	-8	-7	-8.1	-5.3					
TG.02	-7	-8.8	-8.4	-9	-5.3	-7.1	-6.8					
Top-three ligands	Phosphorylation sites of CD24 protein											
	T34	T34	T34									
Suramin	-7.1	-7.1	-7.1									
Rifaximin	-5.2	-5.2	-5.2									
Telmisartan	-4.3	-4.3	-4.3									
Top-three ligands	Phosphorylation sites of PLK1 protein											
	S2	T6	S71	S103	S137	T210	T214	S269				
Suramin	-5.3	-4.6	-7.1	-6.2	-3.4	-4.5	-6.7	-5.6				
Rifaximin	-4.2	-3.5	-5.4	-4.2	-6.1	-5.2	-6.1	-5.2				
Telmisartan	-5.3	-4.3	-6.1	-5.2	-4.2	-4.3	-5.8	-4.8				
Top-three ligands	Phosphorylation sites of UBE2C protein											
	S3	S12	S176									
Suramin	-4.5	-5.3	-3.2									
Rifaximin	-5.3	-4.8	-4.3									
Telmisartan	-5.1	-4.6	-4.1									

Table S6. Binding affinity scores for the proposed top-ranked six ligands with the **ubiquitina**ted PTM sites of hub-proteins

Top six ligands	Uubiquitinated sites of ACACB protein										
	S302	S350	T70	S72	S175	S195	S246	S469	S1360	T2025		

Suramin	-9.2	-7.8	-8.1	-8	-9.2	-7.4	-8.1	-8.1	-9	-7		
Rifaximin	-7.1	-8.5	-8.9	-7.1	-8.5	-6.7	-8.8	-7.9	-8.7	-8.4		
Telmisartan	-8.3	-8.9	-8.1	-7.5	-8.5	-8.1	-5.6	-10	-7.2	-6.9		
Tukysa-Tucatinib	-7.3	-7.6	-7.1	-7.9	-8.4	-7.3	-9.1	-8.3	-10.2	-8.2		
Lynparza-Olaparib	-7	-8.8	-8.4	-9	-7.2	-6.9	-11.1	-8	-8	-12.1		
TG.02	-7	-8.1	-5.3	-6.9	-8.2	-7.5	-8.1	-8.1	-9	-9.1		
**Top six ligands**	**Uubiquitinated sites of CD36 protein**											
	S302	S350										
Suramin	-8.8	-8.2										
Rifaximin	-8.5	-7.3										
Telmisartan	-7.1	-9.5										
Tukysa-Tucatinib	-7.2	-8.2										
Lynparza-Olaparib	-8.5	-5.6										
TG.02	-12.1	-6.2										
**Top six ligands**	**Ubiquitination sites of COL10A1 protein**											
	T61	S87	S381	S384	S471	S533	T547					
Suramin	-7.7	-7.1	-8.4	-6.8	-8	-7	-8.1					
Rifaximin	-8.1	-9.2	-7.3	-8.4	-7.7	-8.3	-8.5					
Telmisartan	-12.1	-8.4	-9	-5.3	-8.1	-8.1	-7.4					
Tukysa-Tucatinib	-8.4	-9	-7.5	-6.8	-8.9	-5.6	-9.1					
Lynparza-Olaparib	-8.1	-8	-8.3	-7.6	-8	-12.1	-8					
TG.02	-12.1	-7.6	-9.5	-8	-7.6	-7.9	-7.8					
**Top six ligands**	**Ubiquitination sites of COL11A1 protein**											
	S50	S134	S240	T358	S370	T659	S1058	T1067	S1481	T1558	S1737	

Suramin	-8.3	-8.9	-7.2	-7.6	-9.1	-12.1	-11.1	-8.2	-12.1	-6.2	-5.6	
Rifaximin	-8.1	-8.4	-7.7	-8.4	-7.1	-8.9	-8.9	-8.7	-9	-7.5	-10.2	
Telmisartan	-8.4	-8.3	-8.1	-8.4	-7.5	-9.2	-7.1	-7.9	-7.6	-8	-7.9	
Tukysa-Tucatinib	-7.2	-6.6	-7.6	-9.5	-9.1	-8.2	-7.5	-9.5	-9	-7.8	-6.2	
Lynparza-Olaparib	-8.7	-8.2	-8.4	-6.8	-11.1	-6.9	-6.2	-8.1	-6.8	-7	-8.1	
TG.02	-12.1	-9.1	-7.9	-8.4	-8.1	-13.1	-7.5	-7.9	-8.4	-7.9	-8.4	
**Top six ligands**	**Ubiquitination sites of PDK4 protein**											
	S10	S13	S33	S106	S222	T316	S390					
Suramin	-7.8	-7.3	-10.2	-9.1	-6.8	-8.1	-9.1					
Rifaximin	-7	-9.5	-8	-5.6	-8.8	-9.1	-8.7					
Telmisartan	-7.3	-7.9	-7.8	-7.7	-8.9	-7.1	-8.8					
Tukysa-Tucatinib	-9.5	-9.2	-7	-7.5	-7.3	-8.4	-7.7					
Lynparza-Olaparib	-11.1	-6.2	-7.2	-8.4	-6.6	-9.5	-8					
TG.02	-8.1	-8.2	-8.3	-8.5	-12.1	-8.3	-7.9					
Top-three ligands	Ubiquitination sites of CD24 protein											
	K56	K81										
Suramin	-6.4	-6.1										
Rifaximin	-5.1	-4.9										
Telmisartan	-5.7	-5.3										
Top-three ligands	Ubiquitination sites of PLK1 protein											
	K9	K19	K38	K178								
Suramin	-7.0	-6.8	-4.5	-5.2								
Rifaximin	-6.2	-6.4	-5.1	-4.6								
Telmisartan	-6.4	-5.3	-4.7	-3.9								
Top-three ligands	Ubiquitination sites of UBE2C protein											

	K18	K61	K119	K172								
Suramin	-5.3	-4.3	-6.3	-5.8								
Rifaximin	-5.2	-5.3	-5.5	-6.1								
Telmisartan	-4.2	-4.6	-5.1	-5.3								
